# 

**Published:** 1872-11

**Authors:** 


### INDEX OF SUBJECTS.

### ORIGINAL COMMUNICATIONS.

A Dressing for Fracture........................................ 651.
Electro-Magnetism, etc......................................... 652.
Correspondence,................................................ 654.
Foreign Correspondence,........................................ 656.
Selections—Kerosene Oil, etc.,................................. 665.

### EXTRACTS AND GLEANINGS.

Indigestion and its Management, 668; Hydrocele Cured by Carbolic-Acid Injections, 675; Dr. Richardson's Treatment of Diabetes—Treatment of Hæmorrhoids, 676; SECOND PART—Spinal Irritation, 677; Lithotrity in Children—Vomiting and Salivation, 679; The use of Acetate of Lead, etc.,—Morphia and Chloral in Combination, 680; Treatment of Melancholy—Temperature in Diseases, 681; Treatment of So-called Hysterical Joint—Surgical Treatment of Anasarca, 682; Anodyne Alterant—Antidote for Arsenic, 681; Nitrate of Silver in Bed-Sores—Houston's Operation, 682; Abortive Treatment of Gonorrhœa—Dry Earth as a Surgical Dressing—Cholera Remedy, 683; Injection of air, etc.—Treatment of Burns and Scalds, 684; Treatment of Gleet—Quinine in Cholera, 685; Fracture of Base of Skull—Excision of the Hip-Joint, 686; Carrots versus Tænia—Chloral as a Local Application, 687; Epithelioma—Effects of Bromide of Potassium, 688; Character and Treatment of Malignant Scrofulous Angina—Injection for Gonorrhea—Prevent Stains from Iodine, 689; Cerebro-Spinal Meningitis — Phagedenic Chancre, 690; Concentrated Emulsion of Almonds—Local Application of Sulphate of Iron, etc., 691, Seneca Root—Danger from Gutta-Percha Catheters—New Styptic, 692; Elixir Quiniæ Ferri et Strychniæ Phosphatis—External use of Fuming Nitric Acid—Morphia in Diabetes, 693; Emmenagogue Formula—Grafting with Rabbit Skin, 694; Action of Soda—Liquid Shampoo—Cure for Sleep Walking, 695; Prunin—Oil of Stillinger—New Reagent for Blood, 696; Use of Diluted Pyroligneous Acid as a Gargle—Secondary Syphilis—Morphia and Chloral, 697; The agency of Mind in Disease—Oil of Staphysagria, 698; New Method of Nourishing by the Rectum—Antacid for Heartburn—Enlarged Testicle, 699; Emulsion of Turpentine Formula for Dyspepsia—Acid of the Tomato, 700; Sick Headache—Rhubarb Bitters—Cholera Infantum, 701.

### EDITORIALS, REVIEWS, ETC.

THIRD PART—Our Original Department—The Late Fire in Boston............ 702.
Kind Words and Suggestions from Friends................................ 703.
Bibliographical........................................................ 705.
Literary Notices....................................................... 710
Advertising Department................................................. 711

